# Development and internal validation of a nomogram based on peripheral blood inflammatory markers for predicting prognosis in nasopharyngeal carcinoma

**DOI:** 10.1002/cam4.7135

**Published:** 2024-03-29

**Authors:** Jing Lai, Peixin Lin, Jiafeng Zhuang, Zhiwei Xie, Hechao Zhou, Donghong Yang, Zihong Chen, Danxian Jiang, Jing Huang

**Affiliations:** ^1^ Department of Head and Neck Oncology Affiliated Hospital of Guangdong Medical University Zhanjiang Guangdong China

**Keywords:** inflammatory markers, nasopharyngeal carcinoma, nomogram, prognostic model, TNM staging system

## Abstract

**Background:**

Inflammatory markers, including the product of neutrophil count, platelet count, and monocyte count divided by lymphocyte count (PIV) and the platelet‐to‐white blood cell ratio (PWR), have not been previously reported as prognostic factors in nasopharyngeal carcinoma (NPC) patients. In order to predict overall survival (OS) in NPC patients, our goal was to create and internally evaluate a nomogram based on inflammatory markers (PIV, PWR).

**Methods:**

A retrospective study was done on patients who received an NPC diagnosis between January 2015 and December 2018. After identifying independent prognostic indicators linked to OS using Cox proportional hazards regression analysis, we created a nomogram with the factors we had chosen.

**Results:**

A total of 630 NPC patients in all were split into training (*n* = 441) and validation sets (*n* = 189) after being enrolled in a population‐based study in 2015–2018 and monitored for a median of 5.9 years. In the training set, the age, PIV, and PWR, selected as independent predictors for OS via multivariate Cox's regression model, were chosen to develop a nomogram. Both training and validation cohorts had C‐indices of 0.850 (95% confidence interval [CI]: 0.768–0.849) and 0.851 (95% CI: 0.765–0.877). Furthermore, compared with traditional TNM staging, our nomogram demonstrated greater accuracy in predicting patient outcomes. The risk stratification model derived from our prediction model may facilitate personalized treatment strategies for NPC patients.

**Conclusion:**

Our findings confirmed the prognostic significance of the PWR and PIV in NPC. High PIV levels (>363.47) and low PWR (≤36.42) values are associated with worse OS in NPC patients.

## 1 INTRODUCTION

Nasopharyngeal carcinoma (NPC) is a type of cancer that develops from the epithelial cells that line the nasopharynx. With the advent of intensity‐modulated radiotherapy (IMRT), among patients with locally advanced NPC, the overall 5‐year survival rate can be higher than 80%, whereas the 10‐year survival rate is closer to 70%.^1^ The most important method for classifying patients with NPC based on their treatment options and assessing their response to treatment is still the tumor, node, metastasis (TNM) staging system, which is based on anatomical characteristics. The TNM staging system is constantly improving with advances in imaging diagnosis technology, radiotherapy, and chemotherapy.^2^, ^3^ However, due to the biological heterogeneity of tumors, clinical results for patients receiving comparable treatment and at the same TNM stage may differ significantly. Therefore, it is critical to identify effective prognostic indicators to formulate individualized treatment strategies aimed at prolonging patient survival time and improving their quality of life.

Numerous studies have been conducted to improve the accuracy of the TNM staging system by including biomarkers. For example, one reliable biomarker for NPC screening, diagnosis, prognosis, and therapy monitoring is plasma Epstein–Barr virus (EBV) DNA.^4^, ^5^ However, the lack of standardized quantitative analysis and high price have hampered the widespread adoption of EBV DNA, although international guidelines, including the National Comprehensive Cancer Network (NCCN) and the European Society of Medical Oncology (ESMO), have recommended that plasma EBV DNA be included in clinical diagnosis.^6^ Other biomarkers, such as circulating tumor cells (CTCs), methylation markers, and miRNAs, have also demonstrated significant prognostic value and potential clinical application in NPC. However, their implementation in clinical practice is challenging due to their high cost, variations in detection techniques, and the presence of tumor heterogeneity in both phenotype and genetic markers.^7^, ^8^, ^9^ In recent years, the development of radiomics based on medical imaging has enabled the characterization of intrinsic biological properties of tumors. However, most algorithms face challenges when transitioning into clinical practice.^10^

Since Rudolf Virchow first discovered leukocytes in tumor tissue in 1863, the link between cancer and inflammation has been gaining attention. Inflammation is a crucial element of the tumor microenvironment, where inflammatory cells can have beneficial effects.^11^ Incorporating inflammatory markers from peripheral blood into the current staging system can serve as a practical and affordable route to predict patients' prognoses with NPC, given their minimal differences in routine testing. According to relevant literature, numerous studies incorporated multiple inflammatory markers, including but not limited to neutrophil‐to‐lymphocyte ratio (NLR), platelet‐to‐lymphocyte ratio (PLR), and lymphocyte‐to‐monocyte ratio (LMR). These indicators give patients with NPC a more complete picture of their immunological response and inflammation.^12^, ^13^ However, the findings of these studies are different. For instance, a study conducted in 2020 by Feng et al. showed that, in univariate analysis, SII was substantially correlated with PFS and OS, but not in multivariate analysis for NLR, PLR, or SII.^14^ Similarly, Yuan et al. in 2022 revealed that SII lacked prognostic value among EBV DNA‐negative population.^15^ Therefore, it is necessary to continue to explore meaningful inflammation indicators. Recently, some novel indicators such as platelet‐to‐leukocyte ratio (PWR) and pan‐immune inflammation value (PIV) have emerged. The PWR has the potential to accurately predict infection and bleeding complications in patients having radical nephrectomy for renal malignant tumors as well as forecast the overall survival (OS) of patients with acute myeloid leukemia.^16^, ^17^ For patients with metastatic colorectal cancer who are receiving biological treatments along with first‐line chemotherapy, the PIV is predictive of both overall survival (OS) and progression‐free survival (PFS). Additionally, it displays the predictive value for PFS and OS in individuals whose pancreatic cancer is locally progressed.^18^, ^19^ Moreover, the PIV is a valid inflammatory marker for predicting OS in patients suffering from breast cancer and cancer‐specific survival (CSS) among those diagnosed with esophageal squamous cell carcinoma (ESCC).^20^, ^21^ However, it has not previously been documented how PWR and PIV affect prognosis in NPC patients. Consequently, this study's goal was to assess PWR and PIV's prognostic significance in NPC. Additionally, we developed and internally validated a nomogram incorporating clinically relevant variables and inflammatory markers to assess its predictive capability.

## 2 MATERIALS AND METHODS

This prediction model study is reported in accordance with the TRIPOD (Transparent Reporting of a Multivariable Prediction Model for Individual Prognosis or Diagnosis) checklist.^22^

### 2.1 Study design and participants

We conducted a retrospective analysis of patients diagnosed with NPC at the Affiliated Hospital of Guangdong Medical University between January 2015 and December 2018. The following were the inclusion criteria: (1) patients diagnosed with NPC based on pathological examination, (2) age ≥ 18 years, and KPS (Karnofsky performance score) ≥70. The following were the exclusion criteria: (1) distant metastasis, (2) nonintensity‐modulated radiotherapy (non‐IMRT), (3) patients with concurrent tumors other than NPC or a history of prior antitumor therapy, (4) acute and chronic inflammatory, infectious or hematological diseases, (5) discontinuation or incomplete treatment, and (6) incomplete clinical or follow‐up data. The study's protocols and techniques adhered to the Declaration of Helsinki's tenets and were approved by the Institutional Review Board of Affiliated Hospital of Guangdong Medical University (number: PJKT2023‐055).

### 2.2 Treatment and data collection

All patients received IMRT. Individuals who did not have distant metastases received either induction chemotherapy and concomitant chemoradiotherapy or radiation alone. According to reports 50 and 62 of the National Committee on Radiation Units and Measurements (ICRU), the prescribed radiation dose for primary nasopharyngeal tumors is 70 Gy or higher, and the radiation dose for cervical metastatic lymph nodes is 60–70 Gy in 30–32 fractions. According to the patient's tolerance, platinum‐based medications were given every 3 weeks during two to three cycles of chemotherapy. A thorough baseline evaluation was performed on each patient, which included a complete medical history, bodily examination, lab testing, and imaging scans. Candidate predictor variables are selected based on evidence derived from the clinical, epidemiological, or predictive model literature.^13^, ^14^, ^16^, ^18^ Therefore, clinically relevant variables were extracted from medical records, including gender, age, tobacco and alcohol consumption habits, TNM stage, IC (induction chemotherapy), CCRT (concurrent chemoradiotherapy) as well as peripheral venous blood parameters (including monocyte, white blood cell, neutrophil, and lymphocyte and platelet counts) recorded within the week preceding treatment. The calculation formulas of inflammatory biomarkers are as described below: PWR = platelet count (10^9^/L)/white blood cell count (10^9^/L); PIV = neutrophil count (10^9^/L) × monocyte count (10^9^/L) × platelet count (10^9^/L)/lymphocyte count (10^9^/L).

### 2.3 Follow‐up

Patients were followed up every 3 months for the first 3 years following treatment completion, every 6 months for the subsequent 4–5 years, and once a year after that. During the follow‐up period, patients underwent physical examination, laboratory testing, and imaging, including palpation of cervical lymph nodes, routine blood tests, biochemical tests, nasal endoscopy, head and neck magnetic resonance imaging (MRI), chest and abdominal computed tomography (CT), as well as whole‐body bone scanning (WBS). The study's main outcome was OS, which is the amount of time that passes between the pathology diagnosis of NPC and either the final follow‐up date (which was April 30, 2023) or the patient's death from any cause.

### 2.4 Statistical analysis

Based on a sample size estimation method developed specifically for clinical predictive models by Richard et al., we use the pmsampsize package in the R language to calculate the minimum sample size we need (Figure S1).^23^ In this study, 705 patients with NPC diagnoses in total were included based on the predetermined inclusion criteria. Among them, 21 patients had incomplete clinical or follow‐up information and were therefore prevented from being included in the analysis due to their limited number of missing cases. The patients were split into training and validation sets using a 7:3 randomization procedure. By calculating the area under the curve (AUC) and using the characteristics of receiver operation (ROC) analysis, the best cutoff values for inflammatory markers (PWR and PIV) were identified. The multivariate COX regression analysis then included the factors that had *p* < 0.05 in the univariate analysis. Ultimately, the nomogram was constructed using independent prognostic variables with *p* < 0.05. The constructed nomogram was evaluated using the C‐index, calibration curves were generated to measure the concordance between observed and predicted survival probabilities, and the net benefit and predictive performance of the nomogram were assessed using decision curve analysis (DCA), net reclassification index (NRI), and composite discriminant improvement index (IDI). Additionally, the column graph was used to establish the patients' overall risk score. Depending on the critical value determined by survival ROC curve analysis, the patients were classified as either high‐risk or low‐risk. The Kaplan–Meier method and Log‐rank test were used to evaluate the significance of survival differences among risk groups. In addition, the total risk score of patients was determined using the nomogram. Subsequently, patients were then separated into groups based on their level of risk, low and high, using the crucial value that was discovered by survival ROC curve analysis. The Kaplan–Meier method and the Log‐rank test were utilized to evaluate the significance of survival differences between groups. A *p*‐value of <0.05 was regarded as statistically significant. The data analysis was conducted using SPSS software (version 26) and R software (version 4.3.1).

## 3 RESULTS

### 3.1 Baseline characteristic

**FIGURE 1:**
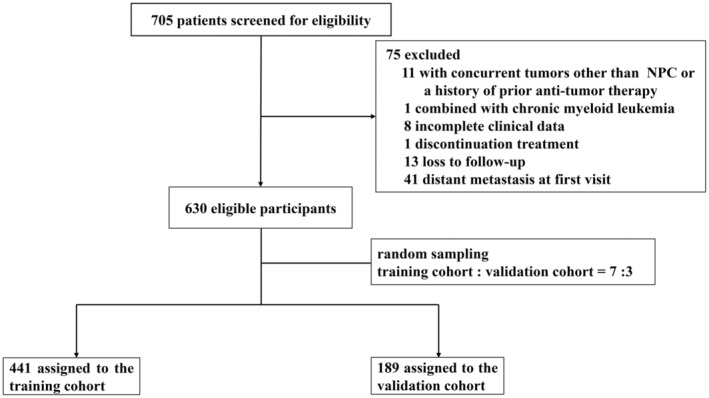
Formation of the derivation training and internal validation cohorts with nasopharyngeal carcinoma. NPC, nasopharyngeal carcinoma.

The enrollment of 630 individuals with NPC diagnoses overall was included according to inclusion and exclusion standards and then randomly assigned 7:3. Specifically, the training set included 441 patients, and the validation set was composed of the remaining 189 cases (Figure 1). In the training cohort, 324 male participants (73.47%) and 117 female participants (26.53%) were included, with a median age of 49 years. The follow‐up period was 71.0 months on average (range = 4.0–104.0 months), and 18.59% of cases (82 persons) had a mortality rate. At the 1‐, 3‐, and 5‐year follow‐up calls, the overall survival rates were determined to be 97.96%, 90.93%, and 84.81%, respectively. However, 47 (24.87%) female and 142 (75.13%) male participants made up the validation set. The participants had a median follow‐up time of 70.0 months (range = 6.0–94.0 months) and a median age of 49 years. A total of 23.28% (44 patients) experienced mortality during the study period. It was discovered that the overall survival rates at 1, 3, and 5 years were 98.94%, 89.95%, and 78.31%, respectively. All patients were restaged using AJCC8th (American Joint Committee on cancer eighth edition). Their baseline characteristics are summarized in Table 1.

**TABLE 1:** Baseline characteristics of study population (*n* [%]).

Characteristic	Training cohort (*n* = 441)	Validation cohort (*n* = 189)	*p*
Age (years)			
≤55	314 (71.20)	137 (72.49)	0.743
>55	127 (28.80)	52 (27.51)	
Gender			
Female	117 (26.53)	47 (24.87)	0.663
Male	324 (73.47)	142 (75.13)	
Drinking			
Yes	59 (13.38)	22 (11.64)	0.550
No	382 (86.62)	167 (88.36)	
Smoking			
Yes	99 (22.45)	54 (28.57)	0.101
No	342 (77.55)	135 (71.43)	
Family history			
Yes	41 (9.30)	18 (9.52)	0.929
No	400 (90.70)	171 (90.48)	
IC			0.895
Yes	355 (80.50)	153 (80.95)	
No	86 (19.50)	36 (19.05)	
CCRT			
Yes	402 (91.16)	170 (89.95)	0.630
No	39 (8.84)	19 (10.05)	
Chinese 2008 T stage			0.542
T1	42 (9.52)	15 (7.94)	
T2	117 (26.53)	48 (25.40)	
T3	147 (33.33)	66 (34.92)	
T4	135 (30.61)	60 (31.75)	
Chinese 2008 N stage			0.080
N0	28 (6.35)	17 (9.00)	
N1	110 (24.94)	62 (32.80)	
N2	224 (50.79)	84 (44.44)	
N3	79 (17.91)	26 (13.76)	
Chinese 2008 stage			0.539
I	9 (2.04)	3 (1.59)	
II	47 (10.66)	23 (12.17)	
III	189 (42.86)	84 (44.44)	
IV	196 (44.44)	79 (41.80)	
AJCC T stage			0.427
T1	39 (8.84)	14 (7.41)	
T2	121 (27.44)	46 (24.34)	
T3	147 (33.33)	70 (37.04)	
T4	134 (30.39)	59 (31.22)	
AJCC N stage			0.148
N0	25 (5.67)	18 (9.52)	
N1	115 (26.08)	56 (29.63)	
N2	223 (50.57)	90 (47.62)	
N3	78 (17.69)	25 (13.23)	
AJCC8th stage			0.415
I	7 (1.59)	3 (1.59)	
II	52 (11.79)	23 (12.17)	
III	189 (42.86)	88 (46.56)	
IV	193 (43.76)	75 (39.68)	

Abbreviations: AJCC8th, American Joint Committee on cancer eighth edition; CCRT, concurrent chemoradiotherapy; IC, induction chemotherapy; N, node; T, tumor.

### 3.2 The optimal cutoff values for PIV and PWR

**FIGURE 2:**
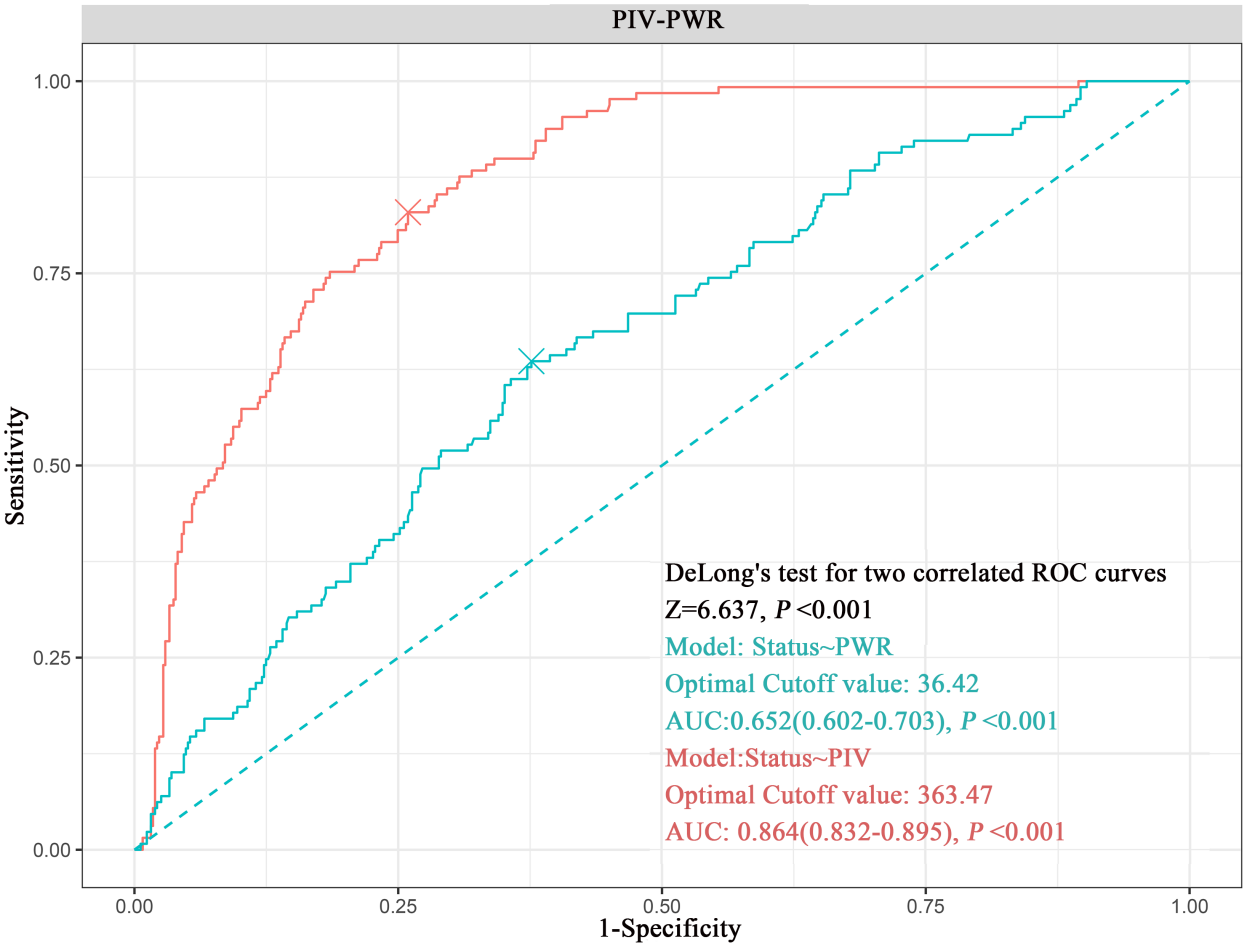
The optimal cutoff values of PWR and PIV. AUC, area under the curve; PIV, pan‐immune‐inflammatory value; PWR, platelet‐to‐white blood cell ratio; ROC, receiver operating characteristic.

The predictive power of inflammatory markers (PIV and PWR) was evaluated using ROC curves. The results demonstrated that PIV and PWR exhibited certain predictive values. With comparable area under the curve (AUC) values of 0.653 and 0.863, the ROC curve analysis revealed that the best cutoff points of PWR and PIV were obtained to be 36.42 (*p* < 0.001) and 363.47 (*p* < 0.001), respectively (Figure 2).

### 3.3 Independent predictors for nasopharyngeal carcinoma

The candidate variables for the prediction model were chosen to identified risk factors (Figure S2) and clinically significant factors (Table 2). The Cox univariate analysis showed that age, PIV, and PWR were identified as significant risk factors for OS in patients with NPC (*p* < 0.05). Due to the high correlation between the PIV and PWR variables, the multicollinearity test (0 < VIF [variance inflating factor] <5) was performed. Age, PIV, and PWR were found to be independent risk variables for OS of NPC patients (*p* < 0.05) according to the Cox multivariate analysis.

**TABLE 2:** Univariate and multivariate Cox regression analyses of the training cohort.

Characteristic	Univariable Cox analysis		Multivariable Cox analysis	
	HR [95% CI]	*p*	HR [95% CI]	*p*
Age (years)				
≤55				
>55	2.874 [1.862–4.435]	<0.001	2.029 [1.306–3.151]	0.002
Gender				
Female				
Male	0.614 [0.350–1.075]	0.088		
Drinking				
Yes	0.917 [0.473–1.778]	0.798		
No				
Smoking				
Yes	1.371 [0.847–2.221]	0.199		
No				
Family history				
Yes	0.572 [0.231–1.414]	0.227		
No				
T stage				
T1				
T2	0.618 [0.247–1.550]	0.305		
T3	1.049 [0.455–2.418]	0.910		
T4	1.644 [0.731–3.694]	0.229		
N stage				
N0				
N1	1.223 [0.360–4.157]	0.747		
N2	1.441 [0.445–4.665]	0.542		
N3	2.451 [0.731–8.222]	0.146		
IC				
Yes	1.845 [0.952–3.577]	0.070		
No				
CCRT				
Yes	2.640 [0.833–8.367]	0.099		
No				
PIV				
≤363.47				
>363.47	10.191 [5.728–18.132]	<0.001	9.711 [5.438–17.340]	<0.001
PWR				
≤36.42				
>36.42	0.390 [0.250–0.608]	<0.001	0.394 [0.250–0.621]	<0.001

Abbreviations: CCRT, concurrent chemoradiotherapy; CI, confidence interval; HR, hazard ratio; IC, induction chemotherapy; N, node; PIV, pan‐immune‐inflammatory value; PWR, platelet‐to‐white blood cell ratio; T, tumor.

### 3.4 Creation and internal validation of the new nomogram for predicting nasopharyngeal carcinoma risk

**FIGURE 3:**
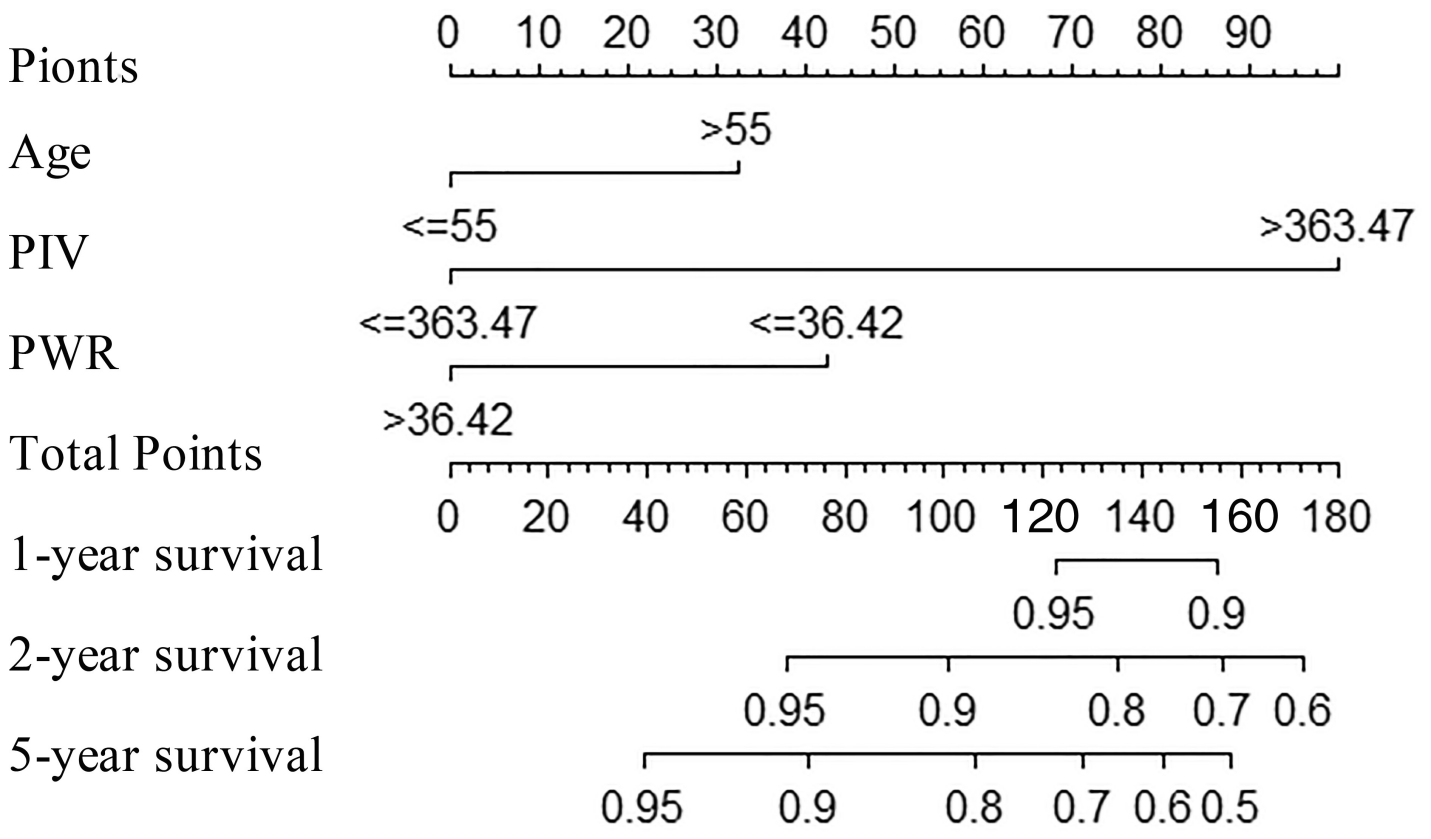
Nomogram for predicting 1‐, 3‐, and 5‐year overall survival. PIV, pan‐immune‐inflammatory value; PWR, platelet‐to‐white blood cell ratio. The total points were derived by aggregating individual scores from the respective variables, with higher scores indicating poorer survival outcomes. Subsequently, the cumulative nomogram points were utilized to predict 1‐, 3‐, and 5‐year survival probabilities.

**FIGURE 4:**
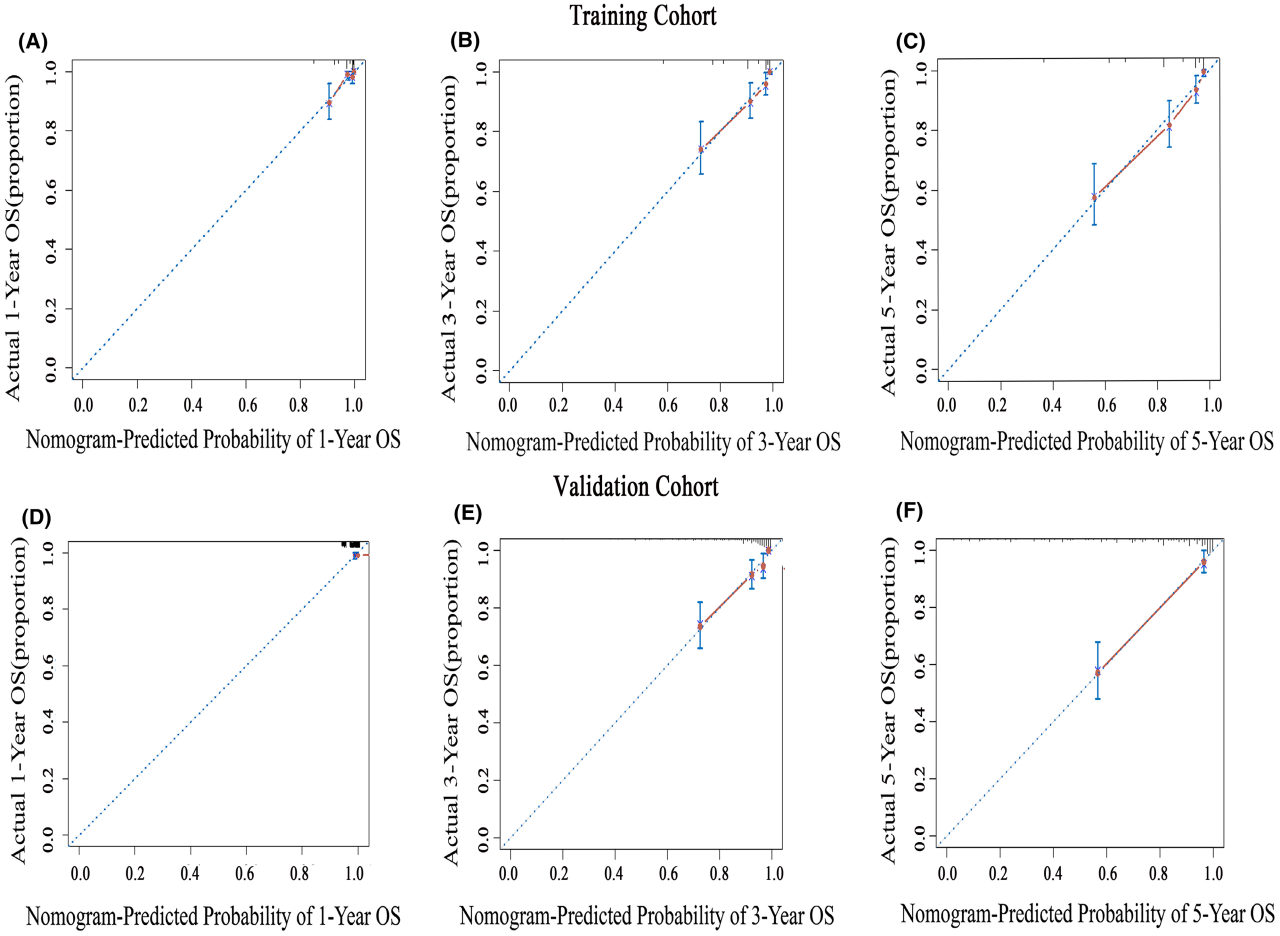
Calibration curve of prediction model at 1‐, 3‐, and 5‐year overall survival. OS, overall survival. Plots of calibration curves to forecast the nomogram's 1‐, 3‐, and 5‐year OS in the training (A–C) and validation (D–F) cohorts.

**FIGURE 5:**
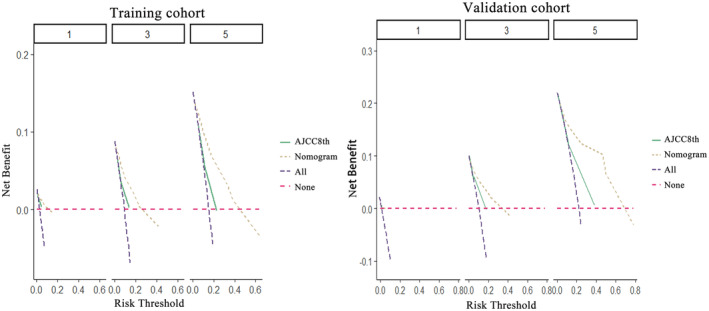
Decision curve analysis of nomogram and the AJCC8th TNM staging system.

A nomogram was constructed using the independent risk factors (age, PIV, and PWR) identified from the aforementioned analysis to accurately predict the OS of NPC patients at 1, 3, and 5 years (Figure 3). A higher total score denotes a worse prognosis. The scores for each variable were ascertained by considering its contribution to the outcome event. The cumulative risk factor assignment scores were then computed in the nomogram. The training set exhibited a C‐index of 0.850 (95% CI: 0.768–0.849), whereas the validation set demonstrated a C‐index of 0.851 (95% CI: 0.765–0.877), indicating good discriminatory capability. The predicted OS rates at 1‐, 3‐, and 5‐year intervals, as indicated by the training and validation sets' calibration curves, exhibited a high level of concordance with the actual observed OS rates (Figure 4). DCA was used to present the clinical validity of the nomogram (Figure 5).

### 3.5 Comparison of the prediction model with the AJCC8th TNM staging system

The nomogram was developed with the AJCC8th TNM staging method to predict the 1‐, 3‐, and 5‐year survival rates for NPC patients. The AJCC8th TNM staging system was not as capable as the prediction model developed in this work, according to the NRI and IDI evaluation (Table 3). Compared to the AJCC8th TNM staging method, the DCA demonstrated that the prediction model had a greater net benefit and clinical validity (Figure 5).

**TABLE 3:** NRI and IDI of the prediction model.

Prediction time	NRI	95% CI	IDI	95% CI
Training cohort				
1‐year survival probability	0.063	[−0.005–0.237]	0.004	[0.002–0.007]
3‐year survival probability	0.175	[−0.046–0.513]	0.017	[0.008–0.025]
5‐year survival probability	0.244	[0.111–0.490]	0.030	[0.016–0.044]
Validation cohort				
1‐year survival probability	0.000	[0.000–0.000]	0.015	[0.008–0.021]
3‐year survival probability	0.157	[−0.237–0.625]	0.112	[0.069–0.155]
5‐year survival probability	0.435	[0.156–0.701]	0.205	[0.142–0.268]

Abbreviations: CI, confidence interval; IDI, integrated discrimination improvement; NRI, net reclassification improvement.

### 3.6 Construction and internal validation of risk stratification model

**FIGURE 6:**
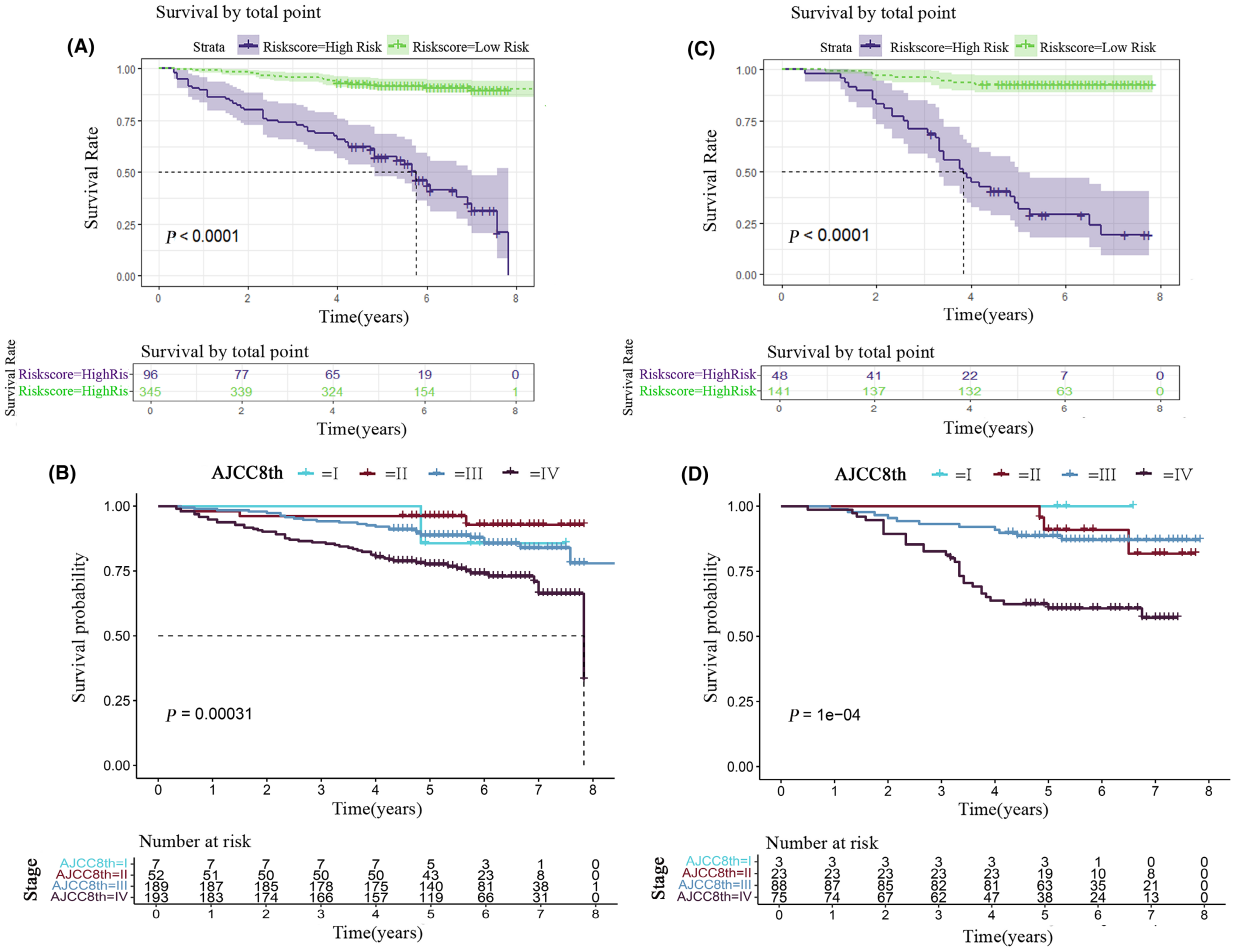
Kaplan–Meier curves demonstrating overall survival in patients of Nasopharyngeal Carcinoma. Kaplan–Meier survival curves for overall survival of the training cohort (A) and validation cohort (B) in different models. AJCC8th staging system was divided into stage I to IV comparisons of the training cohort (C) and validation cohort (D).

We separated the patients into two groups based on their risk scores: low‐risk (score = 0 to 100 scores) and high‐risk (>100 scores) (Figure S3, Table S1). The OS between the low‐risk and high‐risk groups in both the training and validation sets exhibited a significant difference (*p* < 0.0001), indicating that risk stratification effectively enables the identification of patients with a high risk of mortality from NPC (Figure 6). Moreover, the Kaplan–Meier curves displayed a greater difference in risk stratification for nomogram compared to the TNM system.

## 4 DISCUSSION

For the first time, the predictive significance of PIV and PWR in NPC patients is illustrated in this study. The C‐index of OS prediction nomogram (PIV, PWR) based on inflammatory markers was 0.850 (95% CI: 0.768–0.849) and 0.851 (95% CI: 0.765–0.877) in the training and validation cohorts, respectively. This shows that the model is better than the AJCC8th staging approach.

In order to accurately forecast prognosis, it is crucial to develop useful prognostic indicators that might supplement the TNM staging system. Currently, numerous studies have challenged the universal applicability of tumor staging, highlighting the need for personalized approaches. For example, Guo et al. developed an RPA (Robotic Process Automation) model that combines EBV DNA and the TNM staging system to create a unique staging approach, and they performed a retrospective analysis comprising 979 patients with NPC. In the validation cohort (*n* = 550), the new staging system exhibited the AUC of progression‐free survival (PFS) of 0.69 compared to 0.64 for the 8th edition stage of TNM system (*p* = 0.01).^24^ These findings indicate that the newly proposed staging system offers improved risk stratification for patients with NPC and can effectively guide individualized clinical treatment. Li et al. further conducted external validation, and the findings demonstrated that EBV DNA could effectively complement conventional anatomical staging and contribute to prognostic stratification.^25^ At the genetic level, Tang et al. identified 13 out of 137 differentially expressed genes in a multicenter retrospective cohort analysis.^26^ Utilizing a nomogram analysis based on gene expression profiles, N stage, and other indicators, they developed a molecular signature made up of these 13 genes that demonstrated predictive capability for distant metastasis‐free survival (DMFS) in NPC. Furthermore, the accuracy of this prediction increased from 57% to 75%. Additional biomarkers, including microRNA and methylation markers, hold significant value in prognosticating the outcome of patients with NPC.^27^, ^28^, ^29^, ^30^ But the lack of standardization in EBV DNA detection methods and their exorbitant cost have hindered their widespread adoption in clinical practice. Furthermore, radiomics based on medical imaging has the capability to depict the inherent biological characteristics of tumors. Zhang et al. developed a multi‐scale nomogram that integrates clinical characteristics, MRI‐based radiomics features, and pathological images to predict failure‐free survival (FFS) in people suffering from NPC.^31^ Compared with the clinical model, their nomogram demonstrated excellent performance in both internal validation (C‐index: 0.828 and 0.602, *p* < 0.050) and external validation (C‐index: 0.834 and 0.679, *p* < 0.050). However, the use of radiomics in clinical practice is hindered by challenges related to data standardization and model generalization.

Therefore, in order to identify cost‐effective and convenient prognostic indicators for NPC, numerous scholars have employed blood routine tests as an adjunct to TNM staging for forecasting the prognosis of patients suffering from this malignancy. Due to its ability to stimulate blood vessel expansion, cancer cell proliferation, and tumor invasiveness, inflammation can contribute to the onset and progression of cancer, thus necessitating a comprehensive investigation into the tumor microenvironment.^11^, ^32^ Neutrophils, lymphocytes, monocytes, and platelets are crucial constituents of the tumor microenvironment. Patients with malignant tumors often exhibit elevated levels of systemic inflammatory response. During this period, malignant tumors cause a nonspecific inflammatory response that is characterized by a drop in lymphocytes and an increase in platelets and neutrophils. Consequently, the peripheral blood's inflammatory markers prior to malignant tumor treatment can effectively reflect the equilibrium between‐induced inflammation and the immune system's antitumor function, thereby enabling the evaluation of patients' prognosis with malignant tumors. For NPC patients, the existence of lymphocytes is linked to a good prognosis. Nonetheless, tumor‐infiltrating T cells' functional impairment is made worse by soluble substances and metabolic limitations found within the TME.^33^ The lack of stability in a single inflammatory indicator can be overcome by combining multiple inflammatory cells, thereby enhancing the prognostic value.

For those with oral and head and neck squamous cell carcinomas, the PIV could be useful in the form of an indicator for prediction, according to reports, but current research on head and neck tumors primarily focuses on laryngeal cancer patients.^34^, ^35^ Furthermore, it is unknown how important PWR is for prognosis in NPC patients. The current research provides preliminary proof of the predictive importance of PIV and PWR in patients with NPC. The majority of previous studies have looked at the connection between these inflammatory indicators and prognosis; neither a nomogram nor a comparison with TNM staging has been taken into account. Xia et al. developed a nomogram; but the data they used for their research came from individuals who received both IMRT and 2DRT between 2006 and 2011.^36^ This research object of this study is all patients suffered from NPC treated with IMRT, which is more in line with the current clinical radiotherapy technology and has more reference value. By preprocessing the data using the log‐rank test, the ideal cutoff value of PIV in this study was found to be 416.6 (Figure S2). However, a practically applicable cutoff value of 363.47 was obtained from the ROC curve analysis (Figure 2). There were differences between the two. Nevertheless, considering the focus on analyzing binary variables in this study, we opted for utilizing the optimal cutoff value derived from the ROC analysis as it yielded more reliable and applicable results. By conducting a comprehensive literature review, it has been observed that the reference values of PIV and PWR vary across different populations and cancer types. For example, to ascertain the effect of preoperative PIV on the prognosis of 294 patients receiving radical surgery for esophageal squamous cell carcinoma, Feng et al. carried out a retrospective investigation.^20^ Utilizing a restricted cubic spline (RCS) model, they determined an optimal critical value for PIV at 308.2. Furthermore, a total of meta‐analyses involving 2953 breast cancer patients investigated the prognostic significance of PIV (including OS, PFS, pCR [pathological complete response]), revealing cutoff values ranging from 205.1 to 438.7.^37^ The optimal PIV cutoff value of 363.47, as determined in this study, falls within the range consistent with above findings. Based on the PWR reference value, Zhao et al. discovered that patients with a PWR value of more than 25.38 had a poor prognosis.^17^ Furthermore, a retrospective analysis of a database containing 6235 localized renal cancer patients who suffered from radical nephrectomy revealed that those in the lowest quintile for PWR (<24.42) were twice as likely to develop postoperative infections compared to those in the highest quintile (>44.55) (OR [odds ratio]: 2.01, 95% CI 1.42–2.86, *p* < 0 0.001) and also had significantly higher risk of death within 30 days after surgery.^14^ In this study, the ideal PWR cutoff value was 36.42, which was similarly in line with the results of the prior articles. While there is still disagreement on the ideal PIV and PWR cutoff values, patients with NPC who have higher PIV and lower PWR levels are independently linked to a worse prognosis. This observation aligns with earlier research on pancreatic cancer, breast cancer, and other cancers.^38^, ^39^, ^40^ Therefore, both PIV and PWR hold promise as novel inflammatory markers for prognostic prediction in NPC patients. To determine the critical value of inflammatory markers, it is imperative to conduct additional studies with robust sample sizes, particularly multicenter randomized controlled trials. Furthermore, a comprehensive analysis encompassing diverse populations and various cancer types from different geographical regions should be undertaken. These efforts will definitely open the door for the use of inflammatory indicators in clinical settings.

The nomogram was developed by independent risk factors (age, PIV, PWR) to generate individual probabilities of clinical events. Internal validation results demonstrated its superior accuracy compared to traditional TNM staging. A high degree of prognostic accuracy was indicated by the statistical analysis, which showed that the C‐index of the training set was 0.850 (95% CI: 0.768–0.849) and that of the validation set was 0.851 (95% CI: 0.765–0.877). Furthermore, the majority of pertinent literature utilized the C‐index to compare prognostic models and TNM staging systems. For instance, Li et al. developed a nomogram incorporating inflammatory biomarkers (LMR), TNM staging, and other indicators, demonstrating superior performance of the nomogram (C‐index 0.800) compared to TNM staging alone (C‐index 0.672).^41^ In a similar vein, Zhao and colleagues created a nomogram by employing inflammatory markers including LMR and NLR.^42^ The training cohort exhibited a higher C‐index for predicting OS at 0.717 compared with the validation cohort's C‐index of 0.688; both values surpassed those obtained using the 8th edition TNM system (training cohort: C‐index = 0.602; validation cohort: C‐index = 0.599). These findings further support that an inflammation marker‐based nomogram serves as a more dependable prognostic tool for OS prediction in patients with NPC. The statistical methods employed in this study involved NRI and IDI statistical analyses. The NRI of this research showed that the survival prediction ability of the nomogram model at 3, 5 years is better than that of the TNM staging system, while the survival prediction ability at 1 year can only indicate noninferiority to TNM staging system. However, the NRI only focuses on the relative performance of the two prediction models at a certain cutoff value and cannot reflect the overall improvement of the model. The IDI shows the overall improvement of the model and can make up for the shortcomings of NRI. The IDI > 0 was obtained in the training and validation cohorts in this study, and we have reason to believe that the prediction model is superior to the TNM staging system in predicting the OS of nasopharyngeal carcinoma patients and has a certain predictive value. Finally, the risk stratification model constructed by the prediction model can conveniently screen out high‐risk nasopharyngeal carcinoma patients and provide certain clues for induction chemotherapy and the dosage should not be easily reduced during treatment. In addition, the frequency of review after treatment should be increased. Of course, this guiding significance needs to be verified clinically. Second, some prospective clinical studies are needed to evaluate this prediction model.

Although our analysis results suggest that the constructed nomogram is valuable for predicting OS of NPC patients, it is critical to recognize some of our study's shortcomings. First, the study was retrospective and susceptible to selection bias. Second, the sample size was limited to a single center and lacked external validation from multiple centers. Third, the disease is a dynamic process. However, our study was limited to pre‐treatment analysis of the blood routine in patients with NPC. This limitation arises from the susceptibility of these patients to bone marrow suppression following radiotherapy and chemotherapy, as well as potential interference caused by Recombinant human granulocyte stimulating factor and thrombopoietin on blood routine results. Consequently, we refrained from further collection and analysis of changes in the blood routine during and after treatment. Fourth, the exclusion of EBV DNA in our study was due to its routine testing being not implemented at our hospital until 2017, resulting in a higher proportion of missing data.

## 5 CONCLUSIONS

In conclusion, both PIV and PWR demonstrate potential for prognosticating OS in patients with NPC. Furthermore, when it came to forecasting the overall survival of NPC patients, the nomogram we created outperformed the conventional TNM staging. Additionally, the risk stratification model derived from this prediction tool conveniently facilitates patient selection for individualized treatment strategies. Thanks to its affordability and ease of use, this prognostic model is a valuable tool for making customized therapy decisions and assessing the prognosis of patients with NPC.

## AUTHOR CONTRIBUTIONS

**Jing Lai:** Data curation (equal); methodology (equal); software (lead); validation (equal); writing – original draft (lead); writing – review and editing (equal). **PeiXin Lin:** Data curation (equal); methodology (equal); software (equal); visualization (equal). **JiaFeng Zhuang:** Formal analysis (equal); investigation (equal); validation (equal); visualization (equal). **Zhiwei Xie:** Conceptualization (equal); data curation (equal); investigation (equal); validation (equal); visualization (equal). **Hechao Zhou:** Investigation (equal); resources (equal); validation (equal). **Donghong Yang:** Data curation (equal); investigation (equal); resources (equal). **Zihong Chen:** Data curation (equal); investigation (equal); resources (equal). **DanXian Jiang:** Funding acquisition (equal); project administration (equal); resources (equal); supervision (equal); writing – review and editing (equal). **Jing Huang:** Funding acquisition (lead); project administration (lead); supervision (lead); validation (equal); visualization (equal); writing – review and editing (lead).

## FUNDING INFORMATION

This research did not receive any funding.

## CONFLICT OF INTEREST STATEMENT

There are no conflicts of interest with regard to the disclosure of research findings, ethics committee members, guardians of the participants, or the investigators in this study.

## ETHICS STATEMENT

The Independent Review Board of the Guangdong Medical University Affiliated Hospital gave its approval to the retrospective study, which was carried out in accordance with the Declaration of Helsinki's standards (number: PJKT2023‐055, April 25, 2023).

## INFORMED CONSENT STATEMENT

This study is retrospective in nature, so patient permission was not required.

## Supporting information


Figures S1–S5.
Table S1.

## Data Availability

The corresponding author can provide the data from this study upon request. Because patient privacy is a concern, the data are not publicly available.

## References

[1] Tian YM , Liu MZ , Zeng L , et al. Long‐term outcome and pattern of failure for patients with nasopharyngeal carcinoma treated with intensity‐modulated radiotherapy. Head Neck. 2019;41(5):1246‐1252. doi:10.1002/hed.25545 30593728

[2] Guo R , Mao YP , Tang LL , Chen L , Sun Y , Ma J . The evolution of nasopharyngeal carcinoma staging. Br J Radial. 2019;92(1102):20190244. doi:10.1259/bjr.20190244 PMC6774596 31298937

[3] Fourati N , Mnejja W , Nouri OZ , Fèssi W , Siala J , Daoud J . Impact pronostique de la nouvelle classification TNM pour les cancers du nasopharynx. Cancer/Radiothér. 2019;23(6–7):826‐827. doi:10.1016/j.canrad.2019.07.091

[4] Chan KCA , Woo JKS , King A , et al. Analysis of plasma Epstein‐Barr virus DNA to screen for nasopharyngeal cancer. N Engl J Med. 2017;377(6):513‐522. doi:10.1056/NEJMoa1701717 28792880

[5] Expert Committee of Nasopharyngeal Cancer Biomarker, Tumor Biomarker Committee of China Anti‐Cancer Association. 2019,11(3):183–193. doi:10.3969/j.issn.1674-5671.2019.03.01

[6] Lee AWM , Lee VHF , Ng WT , et al. A systematic review and recommendations on the use of plasma EBV DNA for nasopharyngeal carcinoma. Eur J Cancer. 2021;153:109‐122. doi:10.1016/j.ejca.2021.05.022 34153713

[7] Wu J , Zhu H , Gao F , Wang R , Hu K . Circulating tumor cells: a promising biomarker in the management of nasopharyngeal carcinoma. Front Oncol. 2021;11:724150. doi:10.3389/fonc.2021.724150 34778039 PMC8588829

[8] Xu Y , Zhao W , Mo Y , et al. Combination of RERG and ZNF671 methylation rates in circulating cell‐free DNA: a novel biomarker for screening of nasopharyngeal carcinoma. Cancer Sci. 2020;111(7):2536‐2545. doi:10.1111/cas.14431 32324312 PMC7385361

[9] Wang S , Claret FX , Wu W . MicroRNAs as therapeutic targets in nasopharyngeal carcinoma. Front Oncol. 2019;9:756. doi:10.3389/fonc.2019.00756 31456943 PMC6700302

[10] Li S , Deng YQ , Zhu ZL , Hua HL , Tao ZZ . A comprehensive review on radiomics and deep learning for nasopharyngeal carcinoma imaging. Diagnostics (Basel). 2021;11(9):1523. doi:10.3390/diagnostics11091523 34573865 PMC8465998

[11] Sansone P , Bromberg J . Environment, inflammation, and cancer. Curr Opin Genet Dev. 2011;21(1):80‐85. doi:10.1016/j.gde.2010.11.001 21144738

[12] Lin Z , Zhang X , Luo Y , Chen Y , Yuan Y . The value of hemoglobin‐to‐red blood cell distribution width ratio (Hb/RDW), neutrophil‐to‐lymphocyte ratio (NLR), and platelet‐to‐lymphocyte ratio (PLR) for the diagnosis of nasopharyngeal cancer. Medicine (Baltimore). 2021;100(28):e26537. doi:10.1097/MD.0000000000026537 34260530 PMC8284718

[13] Yang S , Zhao K , Ding X , Jiang H , Lu H . Prognostic significance of hematological markers for patients with nasopharyngeal carcinoma: a meta‐analysis. J Cancer. 2019;10(11):2568‐2577. doi:10.7150/jca.26770 31258763 PMC6584332

[14] Feng Y , Zhang N , Wang S , et al. Systemic inflammation response index is a predictor of poor survival in locally advanced nasopharyngeal carcinoma: a propensity score matching study. Front Oncol. 2020;10:575417. doi:10.3389/fonc.2020.575417 33363009 PMC7759154

[15] Yuan X , Yang H , Zeng F , et al. Prognostic value of systemic inflammation response index in nasopharyngeal carcinoma with negative Epstein‐Barr virus DNA. BMC Cancer. 2022;22(1):858. doi:10.1186/s12885-022-09942-1 35932022 PMC9356473

[16] Garbens A , Wallis CJD , Bjarnason G , et al. Platelet to white blood cell ratio predicts 30‐day postoperative infectious complications in patients undergoing radical nephrectomy for renal malignancy. Can Urol Assoc J. 2017;11(11):E414‐E420. doi:10.5489/cuaj.4478 29072562 PMC5698019

[17] Zhao S , Pan H , Guo Q , Xie W , Wang J . Platelet to white blood cell ratio was an independent prognostic predictor in acute myeloid leukemia. Hematology. 2022;27(1):426‐430. doi:10.1080/16078454.2022.2055857 35413229

[18] Fucà G , Guarini V , Antoniotti C , et al. The Pan‐immune‐inflammation value is a new prognostic biomarker in metastatic colorectal cancer: results from a pooled‐analysis of the Valentino and TRIBE first‐line trials. Br J Cancer. 2020;123(3):403‐409. doi:10.1038/s41416-020-0894-7 32424148 PMC7403416

[19] Topkan E , Selek U , Kucuk A , Pehlivan B . Low pre‐chemoradiotherapy Pan‐immune‐inflammation value (PIV) measures predict better survival outcomes in locally advanced pancreatic adenocarcinomas. J Inflamm Res. 2022;15:5413‐5423. doi:10.2147/JIR.S385328 36158517 PMC9499729

[20] Feng J , Wang L , Yang X , Chen Q , Cheng X . Clinical utility of preoperative pan‐immune‐inflammation value (PIV) for prognostication in patients with esophageal squamous cell carcinoma. Int Immunopharmacol. 2023 Aug;15(123):110805. doi:10.1016/j.intimp.2023.110805 37591121

[21] Şahin AB , Cubukcu E , Ocak B , et al. Low pan‐immune‐inflammation‐value predicts better chemotherapy response and survival in breast cancer patients treated with neoadjuvant chemotherapy. Sci Rep. 2021;11(1):14662. doi:10.1038/s41598-021-94184-7 34282214 PMC8289916

[22] Collins GS , Reitsma JB , Altman DG , Moons KG . Transparent reporting of a multivariable prediction model for individual prognosis or diagnosis (TRIPOD): the TRIPOD statement. BMJ. 2015;350:g7594. doi:10.1136/bmj.g7594 25569120

[23] Riley RD , Ensor J , Snell KI , et al. Calculating the sample size required for developing a clinical prediction model. BMJ. 2020;368:m441. doi:10.1136/bmj.m441 32188600

[24] Guo R , Tang LL , Mao YP , et al. Proposed modifications and incorporation of plasma Epstein‐Barr virus DNA improve the TNM staging system for Epstein‐Barr virus‐related nasopharyngeal carcinoma. Cancer. 2019;125(1):79‐89. doi:10.1002/cncr.31741 30351466

[25] Li WZ , Wu HJ , Lv SH , et al. Assessment of survival model performance following inclusion of Epstein‐Barr virus DNA status in conventional TNM staging groups in Epstein‐Barr virus‐related nasopharyngeal carcinoma. JAMA Netw Open. 2021;4(9):e2124721. doi:10.1001/jamanetworkopen.2021.24721 34554238 PMC8461502

[26] Tang XR , Li YQ , Liang SB , et al. Development and validation of a gene expression‐based signature to predict distant metastasis in locoregionally advanced nasopharyngeal carcinoma: a retrospective, multicentre, cohort study. Lancet Oncol. 2018;19(3):382‐393. doi:10.1016/S1470-2045(18)30080-9 29428165

[27] Islam KA , Chow LK , Kam NW , et al. Prognostic biomarkers for survival in nasopharyngeal carcinoma: a systematic review of the literature. Cancers (Basel). 2022 Apr 24;14(9):2122. doi:10.3390/cancers14092122 35565251 PMC9103785

[28] Wang T , Wu J , Wu Y , et al. A novel microRNA‐based signature predicts prognosis among nasopharyngeal cancer patients. Exp Biol Med (Maywood). 2021;246(1):72‐83. doi:10.1177/1535370220958680 32941074 PMC7797999

[29] Han B , Yang X , Zhang P , et al. DNA methylation biomarkers for nasopharyngeal carcinoma. PLoS One. 2020;15(4):e0230524. doi:10.1371/journal.pone.0230524 32271791 PMC7144954

[30] Lu S , Yu Z , Xiao Z , Zhang Y . Gene signatures and prognostic values of m6A genes in nasopharyngeal carcinoma. Front Oncol. 2020;11:875. doi:10.3389/fonc.2020.00875 PMC7300221 32596151

[31] Zhang F , Zhong LZ , Zhao X , et al. A deep‐learning‐based prognostic nomogram integrating microscopic digital pathology and macroscopic magnetic resonance images in nasopharyngeal carcinoma: a multi‐cohort study. Ther Adv Med Oncol. 2020;12:1758835920971416. doi:10.1177/1758835920971416 33403013 PMC7739087

[32] Arneth B . Tumor microenvironment. Medicina (Kaunas). 2019;56(1):15. doi:10.3390/medicina56010015 31906017 PMC7023392

[33] Verma NK , Wong BHS , Poh ZS , et al. Obstacles for T‐lymphocytes in the tumour microenvironment: therapeutic challenges, advances and opportunities beyond immune checkpoint. EBioMedicine. 2022;83:104216. doi:10.1016/j.ebiom.2022.104216 35986950 PMC9403334

[34] Yeh CC , Kao HK , Huang Y , et al. Discovering the clinical and prognostic role of Pan‐immune‐inflammation values on Oral cavity squamous cell carcinoma. Cancers (Basel). 2023;15(1):322. doi:10.3390/cancers15010322 36612318 PMC9818418

[35] Guven DC , Erul E , Yilmaz F , et al. The association between pan‐immune‐inflammation value and survival in head and neck squamous cell carcinoma. Eur Arch Otorrinolaringol. 2023;280(5):2471‐2478. doi:10.1007/s00405-022-07804-x 36565325

[36] Li XH , Chang H , Xu BQ , et al. An inflammatory biomarker‐based nomogram to predict prognosis of patients with nasopharyngeal carcinoma: an analysis of a prospective study. Cancer Med. 2017;6(1):310‐319. doi:10.1002/cam4.947 27860387 PMC5269708

[37] Qi X , Qiao B , Song T , et al. Clinical utility of the pan‐immune‐inflammation value in breast cancer patients. Front Oncol. 2023;13:1223786. doi:10.3389/fonc.2023.1223786 37711203 PMC10499041

[38] Lin F , Zhang LP , Xie SY , et al. Pan‐immune‐inflammation value: a new prognostic index in operative breast cancer. Front Oncol. 2022;12:830138. doi:10.3389/fonc.2022.830138 35494034 PMC9043599

[39] Tang F , Dai P , Wei Q , et al. The neutrophil‐to‐monocyte ratio and platelet‐to‐white blood cell ratio represent novel prognostic markers in patients with pancreatic cancer. Gastroenterol Res Pract. 2021;2021:6693028. doi:10.1155/2021/6693028 34122538 PMC8169265

[40] Ligorio F , Fucà G , Zattarin E , et al. The Pan‐immune‐inflammation‐value predicts the survival of patients with human epidermal growth factor receptor 2 (HER2)‐positive advanced breast cancer treated with first‐line Taxane‐Trastuzumab‐Pertuzumab. Cancers (Basel). 2021 Apr 19;13(8):1964. doi:10.3390/cancers13081964 33921727 PMC8073809

[41] Li J , Chen S , Peng S , et al. Prognostic nomogram for patients with nasopharyngeal carcinoma incorporating hematological biomarkers and clinical characteristics. Int J Biol Sci. 2018;14(5):549‐556. doi:10.7150/ijbs.24374 29805306 PMC5968847

[42] Zhao R , Liang Z , Chen K , Zhu X . Nomogram based on inflammatory biomarkers and nutritional indicators for predicting overall survival in locoregionally advanced nasopharyngeal carcinoma. J Inflamm Res. 2022;15:2971‐2981. doi:10.2147/JIR.S366299 35602661 PMC9122053

