# Correction to “Development and internal validation of a nomogram based on peripheral blood inflammatory markers for predicting prognosis in nasopharyngeal carcinoma”Lai J, Lin P, Zhuang J, Xie Z., Zhou H., Yang D., Chen Z., Jiang D., Huang J. Development and internal validation of a nomogram based on peripheral blood inflammatory markers for predicting prognosis in nasopharyngeal carcinoma. Cancer Med. 2024; 13:e7135. doi:10.1002/cam4.7135

**DOI:** 10.1002/cam4.7333

**Published:** 2024-06-20

In Figure 3, the spelling of points in the first line was incorrect. This should be “Points,” not “Pionts,” The scale bar in the first row was not marked with 100. This should be marked 100 in the scale bar in the first row. In addition, the figure notes “2‐year survival” was incorrect. This should be “3‐year survival.”

The revised Figure 3 is shown here:

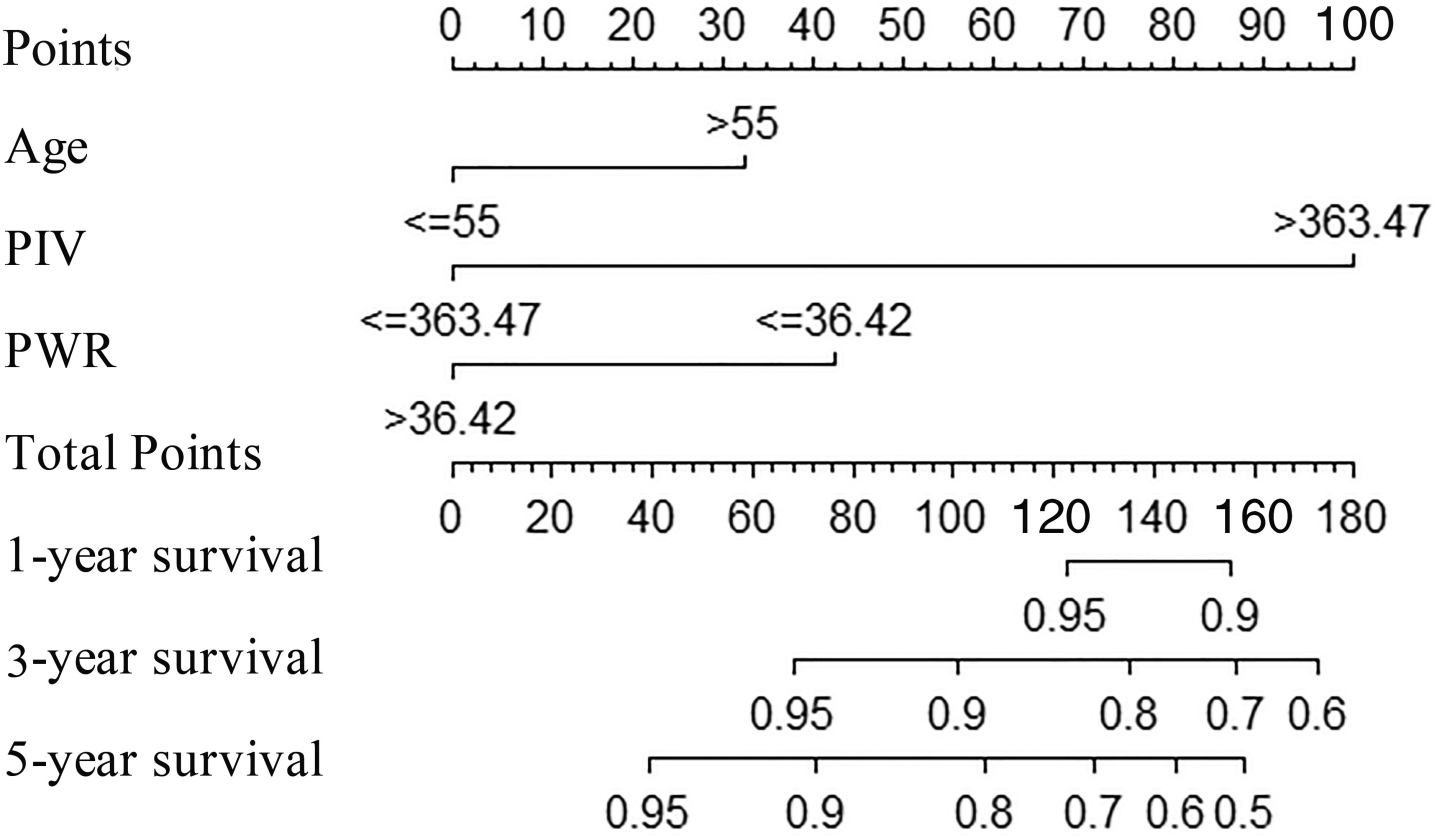

We apologize for these errors.

