# Supplementary material for: Development and internal validation of a nomogram based on peripheral blood inflammatory markers for predicting prognosis in nasopharyngeal carcinoma
Source: Cancer Med. 2024 Mar 29;13(7):e7135. doi: 10.1002/cam4.7135 (PMC10979185; doi:10.1002/cam4.7135)
Supplement: Supplementary file 1 — Figures S1–S5. Table S1. [file CAM4-13-e7135-s001.docx]

Supplementary Material

Development and internal validation of a nomogram based on peripheral blood inflammatory markers for predicting prognosis in nasopharyngeal carcinoma

**Jing Lai^1#^, Peixin Lin^1#^, Jiafeng Zhuang^1^, Zhiwei Xie^1^, Hechao Zhou^1^, Donghong Yang, Zihong Chen^1^****, Danxian Jiang^1^**^*^**, Jing Huang^1^**^*^

^1^ Department of Head and Neck Oncology, Affiliated Hospital of Guangdong Medical University, Zhanjiang, Guangdong, China

^#^Jing Lai and Peixin Lin contributed equally to this work.

*Correspondence: Danxian Jiang, Department of Head and Neck Oncology, Affiliated Hospital of Guangdong Medical University, Zhanjiang, Guangdong, 524000, China. Email:[fyjiangdanxian@163.com](mailto:fyjiangdanxian@163.com); Jing Huang, Department of Head and Neck Oncology, Affiliated Hospital of Guangdong Medical University, Zhanjiang, Guangdong, 524000, China. Email:[huangjing2018@gdmu.edu.cn](mailto:huangjing2018@gdmu.edu.cn)

# Supplementary Figure 1

The minimum sample size required to build a multivariate prediction model of survival outcomes using 10 candidate predictors (such as age, gender, T stage, N stage, AJCC8^th^, drinking, smoking, CCRT, IC, PWR, PIV) was calculated. R^2^ (coefficient of determination) is not found in the literature, consult the solution, in the absence of a specific R^2^ value, you can take 0.15. In addition, in previous studies, the average follow-up time was 5.83 years and the overall event rate was 0.172. We choose a point in time of interest and make predictions using a newly developed 5-year model. Using R language (pmsampsize package), the minimum sample size is 549.


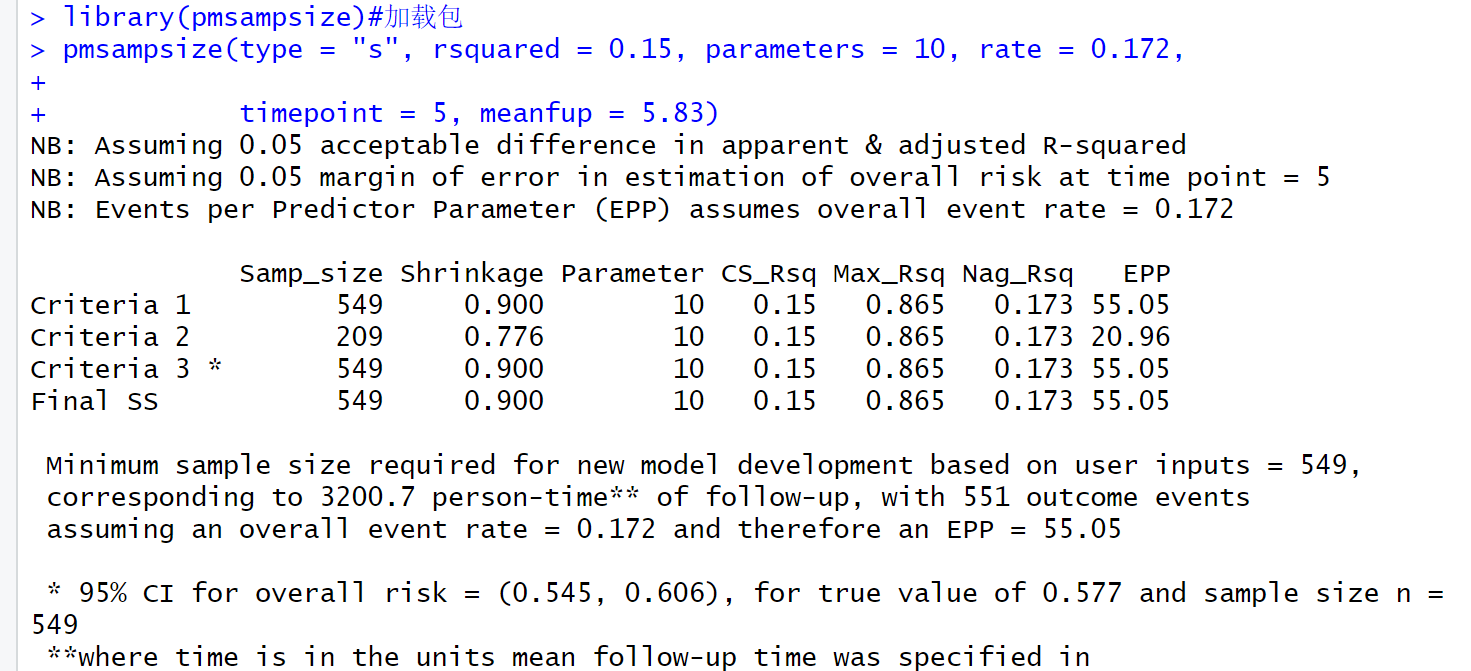


**Supplementary Figure 1:** The calculation of sample size.

**Abbreviations:** R^2^, coefficient of determination; T, tumor; N, node; AJCC8^th^, American Joint Committee on cancer eighth edition; CCRT, concurrent chemoradiotherapy); IC, induction chemotherapy; PWR, platelet-to-white blood cell ratio; PIV, pan-immune-inflammatory value.

# Supplementary Figure 2


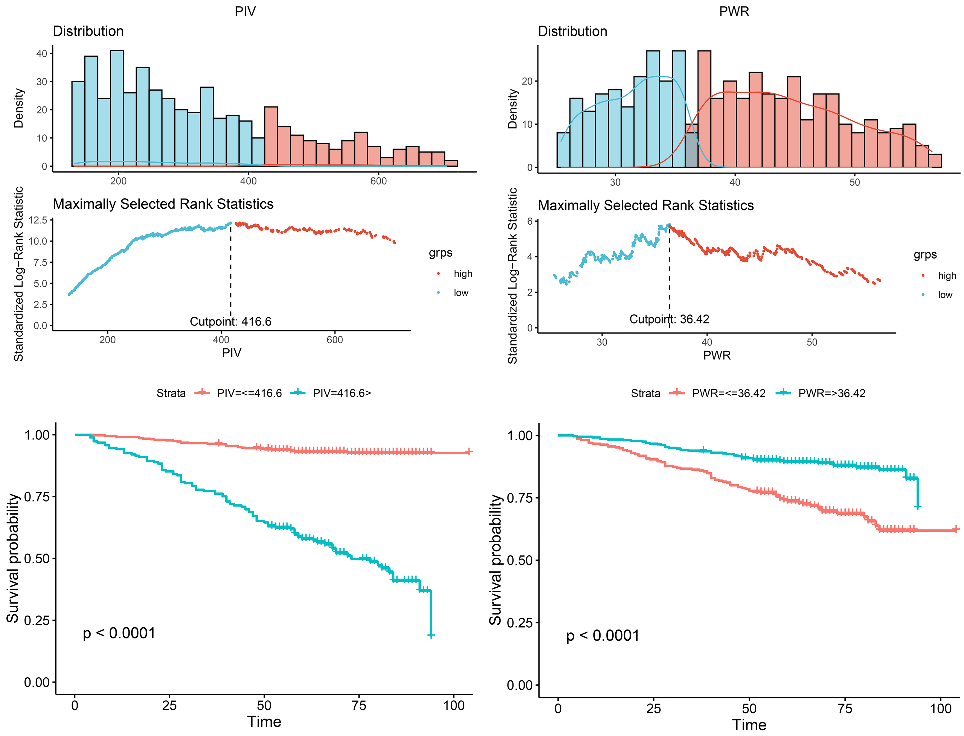


**Supplementary Figure 2:** Risk assessment was conducted for PIV and PWR.

**Notes:** Risk assessment was performed for PIV and PWR, wherein the data were stratified based on the optimal cut-off value, and survival curves were subsequently plotted accordingly.

# Supplementary Figure 3


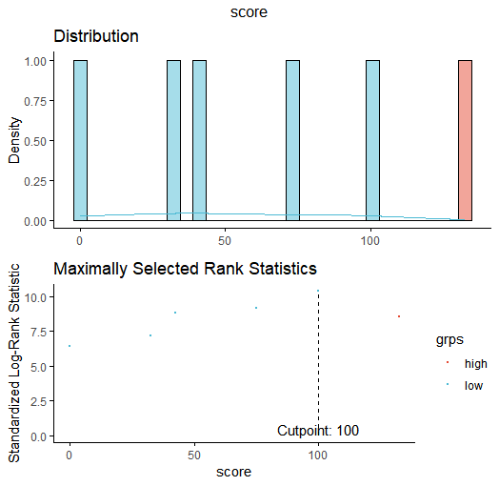


**Supplementary Figure 3:** Risk cutpoint of nomogram.

# Supplementary Figure 4


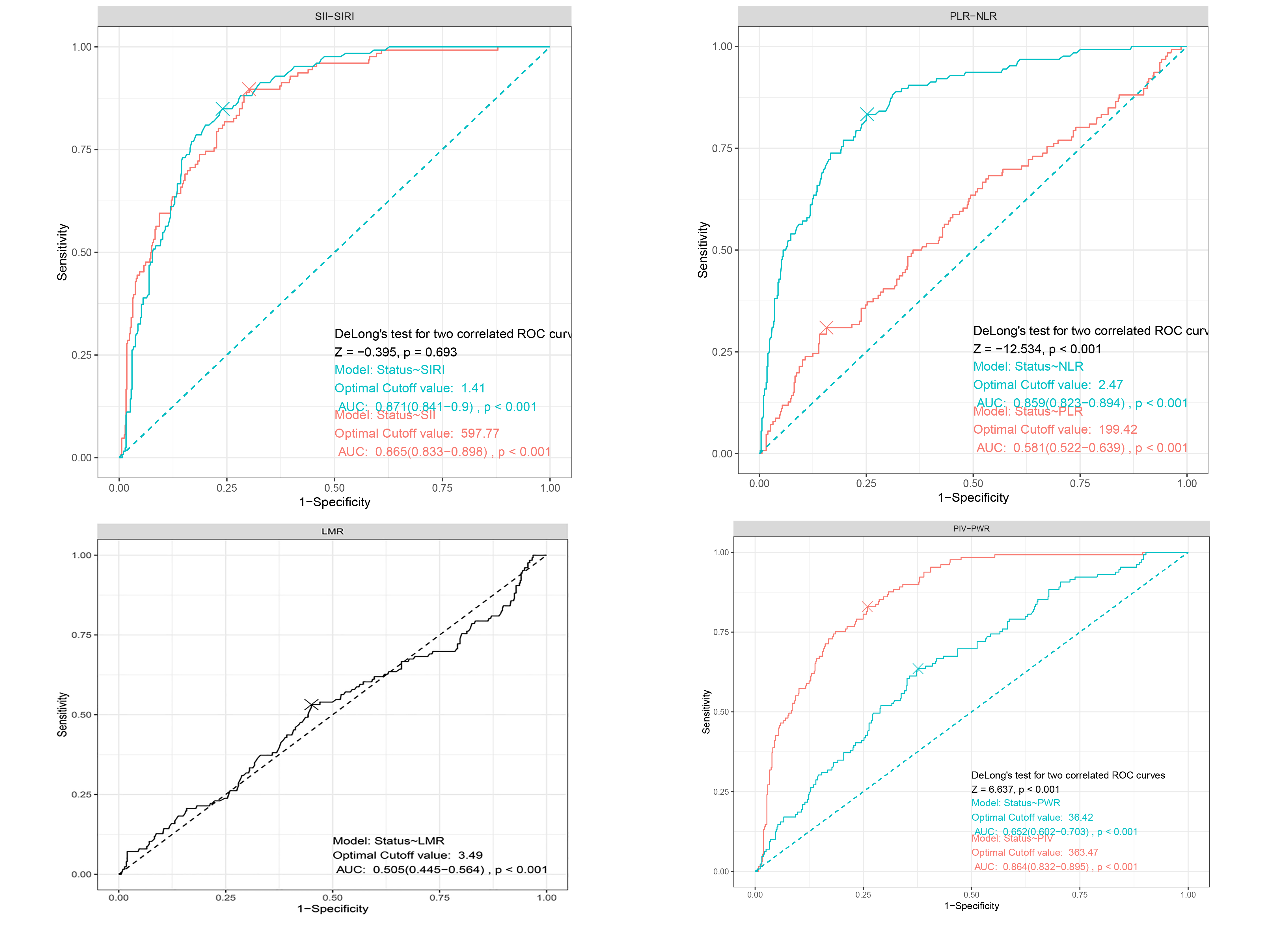


**Supplementary Figure 4:** The area under the curve of inflammatory markers (SII, NLR, SIRI, LMR, PLR, PIV, PWR).

**Abbreviations:** ROC, receiver operating characteristic; AUC, area under the curve; SII, systemic immune-inflammation index; NLR, neutrophil-lymphocyte ratio; PIV, pan-immune-inflammatory value; SIRI, systemic inflammation response index; LMR, lymphocyte-monocyte ratio; PWR, platelet-to-white blood cell ratio; PLR, platelet-lymphocyte ratio.

# Supplementary Figure 5


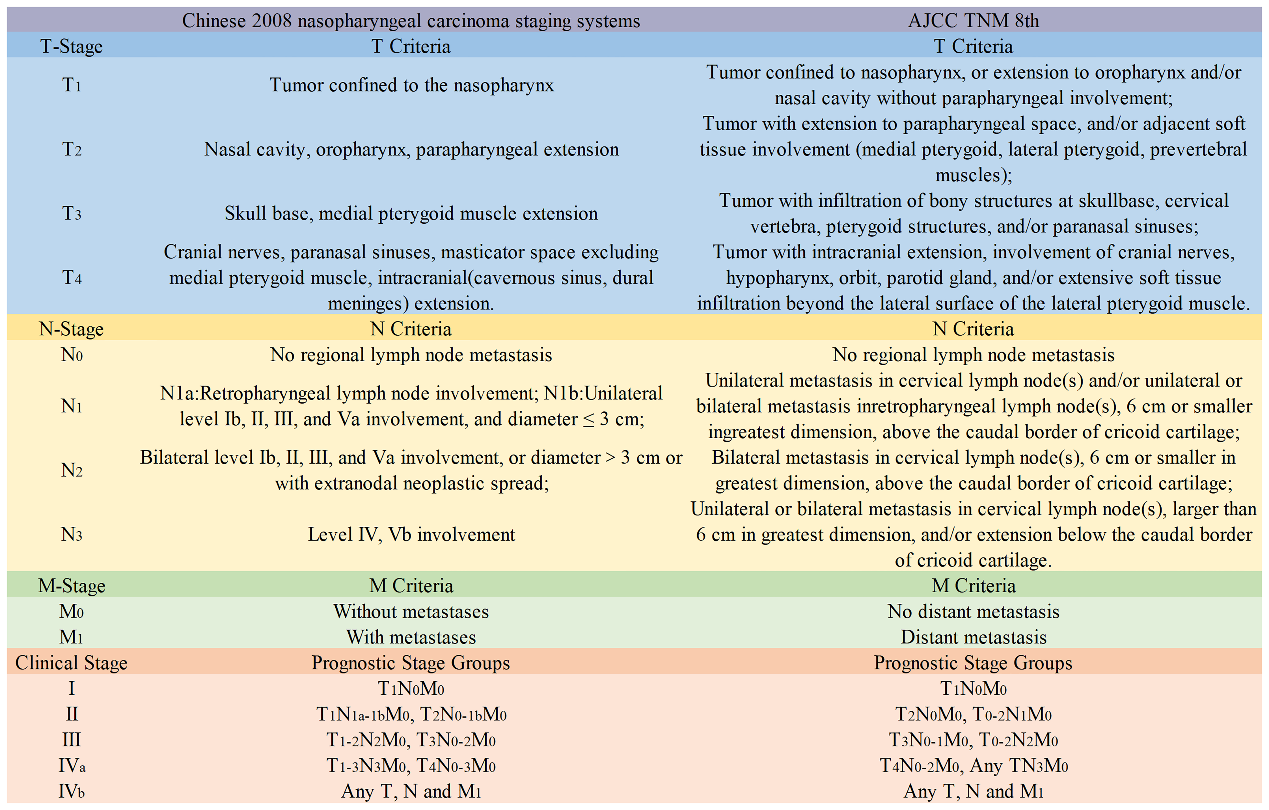


**Supplementary Figure 5:** The comparison chart of Chinese 2008 stage and AJCC8^th^ TNM stage.

# Supplementary Table 1

| **Variables** | **Risk Scores** |  |
| --- | --- | --- |
| **Age（years）**  ≤55  ＞55 | 0  32.56 |  |
| **PIV**  ≤ 363.47  ＞363.47 | 0  100.00 |  |
| **PWR**  ≤ 36.42  ＞36.42 | 42.51  0 |  |
| **1-year OS probability**  0.90  0.95 | 155.00  123.00 |  |
| **3-year OS probability**  0.60  0.70  0.80  0.90  0.95 | 173.00  156.00  135.00  101.00  68.00 |  |
| **5-year OS probability**  0.50  0.60  0.70  0.80  0.90  0.95 | 158.20  144.30  127.90  106.5  72.30  39.50 |  |

**Supplement Table 2:** Risk scores of variables.

**Abbreviations:** PIV, pan-immune-inflammatory value; PWR, platelet-to-white blood cell ratio; OS, overall survival.
